# Incongruent reduction following post-traumatic hip dislocations as an indicator of intra-articular loose bodies: A prospective study of 117 dislocations

**DOI:** 10.4103/0019-5413.73650

**Published:** 2011

**Authors:** K Karthik, SR Sundararajan, J Dheenadhayalan, S Rajasekaran

**Affiliations:** Department of Orthopedic Surgery, James Cook University Hospital, Marton Road, Middlesbrough, United Kingdom; 1 Department of Orthopedics, Traumatology, and Spine Surgery, 313, Mettupalayam Road, Ganga Hospital, Coimbatore, Tamil Nadu, India

**Keywords:** Hip dislocation, intra-articular loose bodies, nonconcentric reduction, postreduction fluoroscopy

## Abstract

**Background:**

Intra-articular loose bodies following simple dislocations can lead to early degeneration. Nonconcentric reduction may indicate retained loose bodies and offer a method to identify patients requiring exploration so that this undesirable outcome can be avoided.

**Materials and Methods:**

One hundred and seventeen consecutive simple dislocations of the hip presenting to the hospital from January 2000 to June 2006 were assessed for congruency after reduction by fluoroscopic assessment of passive motion in the operating room as well as with good quality radiographs. Computerized tomography (CT) scan with 2-mm cuts was done for confirmation of reduction and to identify the anatomy of loose bodies. Patients with nonconcentric reduction underwent open exploration to identify the etiology of the dislocation and for removal of loose bodies. Thomson and Epstein clinical and radiological criteria were used to assess the outcome.

**Results:**

Twelve of the one hundred and seventeen (10%) dislocations had incongruent reduction, which was identified by the break in Shenton’s line and increase in medial joint space in seven patients, increase in the superior joint space in three patients, or increase in the joint space as a whole in two patients. CT scan identified the origin of the osteocartilaginous fragment as being from the acetabulum in six patients, the femoral head in four, and from both in one. One patient had an inverted posterior labrum. Following debridement, congruent reduction was achieved in all patients. At an average follow-up of 5 years (range: 2 years 5 months to 8 years), the outcome as evaluated by Thompson and Epstein clinical criteria was excellent in eleven cases and good in one case; the radiological outcome was excellent in eight cases and good in four cases.

**Conclusions:**

Intra-articular loose bodies were identified by nonconcentric reduction in 12 out of 117 patients with simple hip dislocation. Careful evaluation by fluoroscopy and good quality radiographs are indicated following reduction of hip dislocations.

## INTRODUCTION

Dislocations of the hip are high-energy injuries, with the potential for joint degeneration and long-term disability.^1^ The results following dislocation depend not only on the initial energy of the violence but also on the timing of reduction and the presence or absence of associated fracture.^2^–^4^ Many injuries which appear as simple dislocations may in fact be fracture-dislocations, with the fracture fragments not being easily visualized in the initial radiographs either due to the poor quality of the trauma films or because the small osteocartilaginous fracture fragments are not easily visible in the X-ray.^5^,^6^ These fragments may be entrapped within the joint space following reduction and may accelerate the degenerative process.^1^,^2^,^5^–^7^ Routine postreduction CT scans have shown that in a large number of cases there are intra-articular loose bodies.^4^,^8^,^9^ Entrapment of these loose bodies can be identified in the immediate postreduction period as they may present as incongruent reduction in the immediate postreduction films.^1^,^4^,^8^ The presence of incongruous reduction indicates the entrapment of loose bodies and may form an important indication for open exploration for removal of these loose bodies to prevent degenerative arthritis. We report here the results of our long-term prospective study where 117 simple dislocations of the hip were examined for incongruent reduction by immediate postreduction fluoroscopy and X-rays, the findings of which were compared with postreduction CT scan.

## MATERIALS AND METHODS

**Figure 1:**
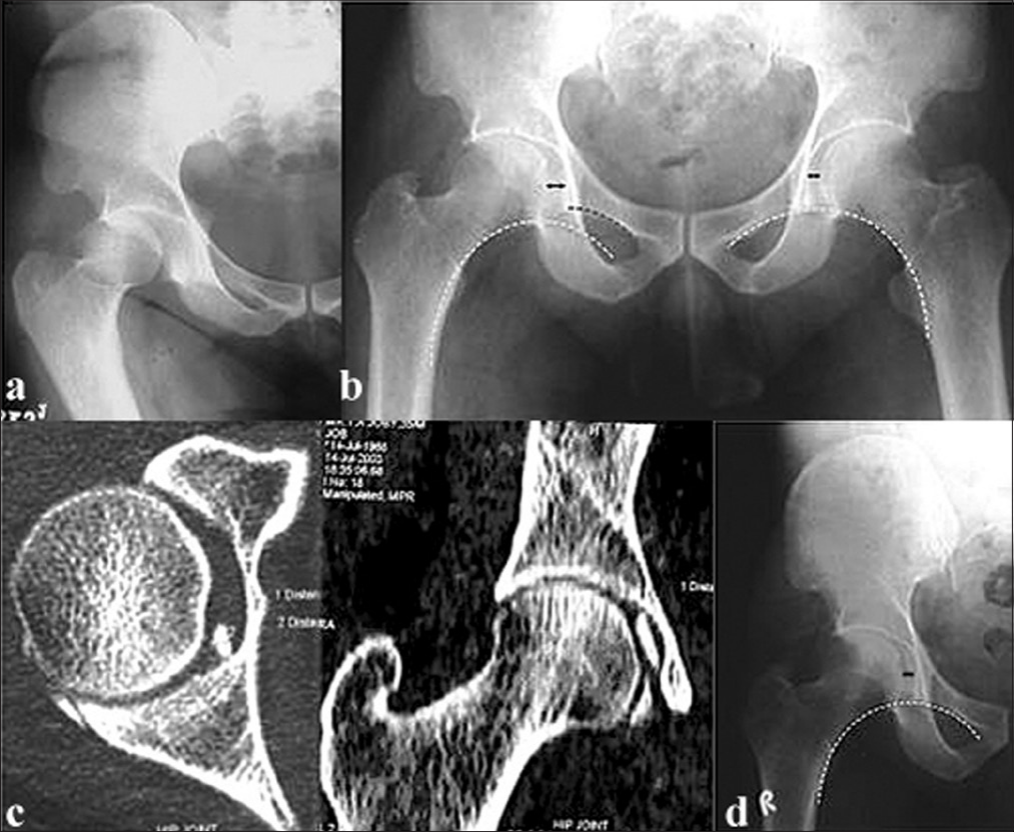
(a) X-ray of right hip joint (an anteroposterior view) showing posterior dislocation of hip in a 44-year-old male. (b) Postreduction X-ray (an anteroposterior view) shows noncongruent reduction: there is a broken Shenton’s line and increase in medial joint space. (c) CT scan shows the presence of an osteochondral fragment on the medial side of the joint. This was removed by open reduction. (d) A postoperative X-ray shows congruent reduction. The origin of the loose body was from the posterior lip of the acetabulum

**Figure 2:**
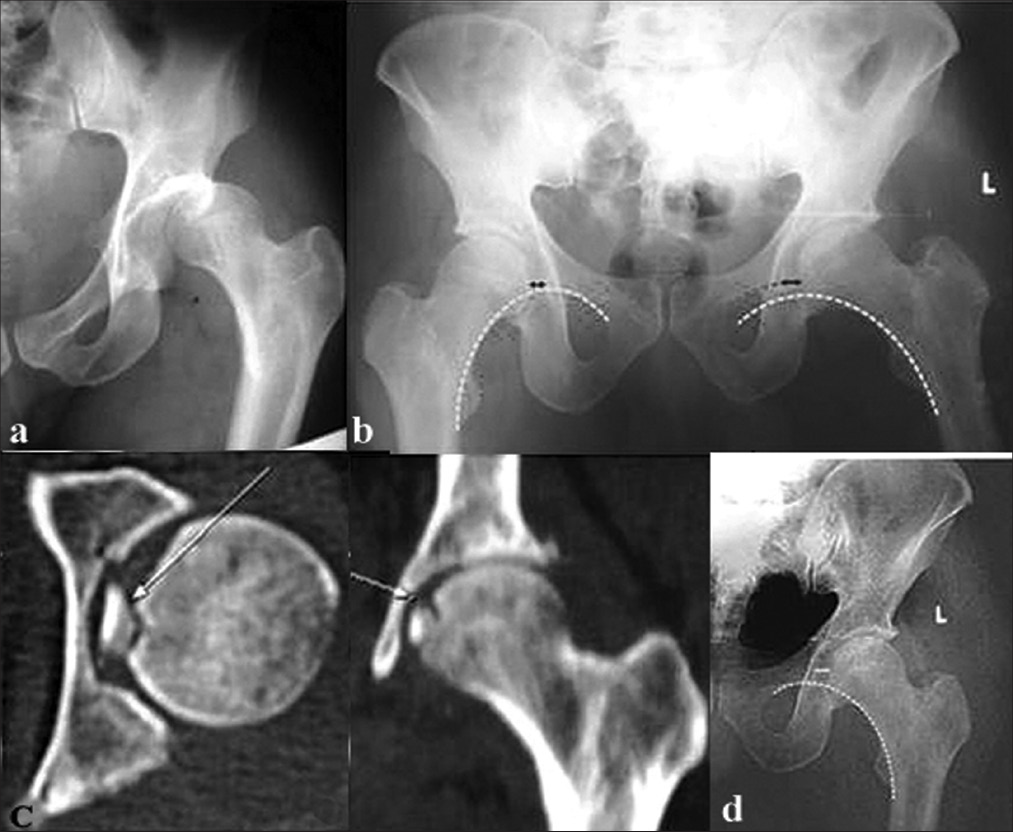
X-ray of left hip joint (an anteroposterior view) in a 53-year-old male showing (a) Posterior dislocation of hip joint. (b) Postreduction X-ray shows noncongruent reduction: a broken Shenton’s line and increase in medial joint space. (c) CT scan showed a noncongruent reduction due to the presence of a large osteochondral fragment from the femoral head. (d) The fragment was excised and the joint reduced, leading to congruent reduction

The study was approved by the institutional review board (IRB), and informed consent was obtained from each patient. During the period between January 2000 and June 2006, 206 hip dislocations in adults were treated in our hospital by the closed method. 89 patients who had obvious fracture-dislocations or irreducible dislocations, as well as those having a fracture of the acetabulum or femoral head leading to fixations, were excluded from the study. The remaining 117 dislocations were diagnosed to have Thomson and Epstein type I fracture^10^ and were reduced by closed methods [Figure 1a and 2a]. Congruency after reduction was assessed immediately in the operating room by fluoroscopic study of passive motion, as well as with radiography (anteroposterior, lateral, obturator, and iliac views) for any break in Shenton’s line, increase joint space when compared to the other normal hip, incongruent joint space, and nonconcentric movement under fluoroscopy [Figure 1b and 2b]. Nonconcentric movement was defined as the movement of the femoral head with a new center of rotation over the concave surface of the acetabulum as compared to the opposite hip. Incongruent joint space was defined as discrepancy between the joint surfaces when compared to the normal hip.

All 117 patients had CT scan with 2-mm cuts for confirmation of reduction and detection of loose bodies. The medial joint space was measured as the distance between the tear drop and the femoral head, the superior joint space as the distance from the highest point in the concave surface of acetabulum to the highest point in the femoral head, and the inferior joint space as the distance between the lowest points of the femoral head and the acetabulum. The measurements in fluoroscopy and X-ray were evaluated by two non-blinded observers and these were confirmed by CT pictures, which were reported by the radiologist.

The patients with incongruent reduction [Table 1] underwent open exploration to achieve a congruent reduction. All cases were operated by the posterior approach. The femoral head was dislocated to extract loose fragments in the anterior and medial side of the joint. Copious irrigation of the joint was done with saline to remove small adherent fragments. Intraoperative postreduction congruency was assessed using fluoroscopy. Postoperatively all the patients were given 2 weeks of rest and allowed to weight bear thereafter according to the level of comfort. The patients were assessed using Thomson and Epstein clinical and radiological criteria^6^ [Table 2] at the 3^rd^, 6^th^, and 12^th^ month, and yearly thereafter, for any arthritic or avascular necrotic changes.

**Table 1:** Patient cohort

Case	Age (in years) / Sex	Cause	Location	Direction	Time interval between injury and reduction (in hours)	Associated injuries	Time interval between injury and loose body extraction
1.	45/M	RTA	R	P	4	Soft tissue injuries R leg	24 hours
2.	52/M	RTA	R	A	3	-	36 hours
3.	36/F	RTA	L	P	5	L patella fracture	24 hours
4.	32/M	RTA	R	P	5	Soft tissue injuries R foot	24 hours
5.	60/M	RTA	R	P	3	L scapula fracture	46 hours
6.	36/M	RTA	R	P	7	-	12 hours
7.	44/M	RTA	R	P	6	-	36 hours
8.	49/M	RTA	R	P	3	-	24 hours
9.	46/M	RTA	R	P	5	-	12 hours
10.	53/M	RTA	L	P	7	-	24 hours
11.	54/M	RTA	R	P	4	R bimalleolar fracture	1 month
12.	27/M	RTA	L	P	8	Soft tissue injuries L ankle	24 hours

M-Male, F-female, RTA-road traffic accident, R-right, L-left, P-posterior, A-anterior

**Table 2:** Thompson and Epstein clinical and roentgenographic criteria^6^

	Clinical criteria	Roentgenographic criteria
Excellent	All of the following: no pain; full range of hip motion; no roentgenographic evidence of progressive changes	All of the following: normal relationship between the femoral head and the acetabulum; normal articular cartilage space; normal density of the head of the femur; no spur formation; no calcification in the capsule
Good	No pain; free motion (75% of normal hip motion); no more than a slight limp; minimum roentgenographic changes	Normal relationship between the femoral head and the acetabulum; minimum narrowing of the cartilage space; minimum deossification; minimum spur formation; minimum capsular calcification
Fair	Any one or more of the following: pain (but not disabling pain); limited motion of the hip; no adduction deformity; moderate limp; moderately severe roentgenographic changes	Normal relationship between the femoral head and the acetabulum. Any one or more of the following: moderate narrowing of cartilage space; mottling of the head, areas of sclerosis, and decreased density; moderate spur formation; moderate to severe capsular calcification; depression of the subchondral cortex of the femoral head
Poor	Any one or more of the following: disabling pain; marked limitation of motion or adduction deformity; redislocation; progressive roentgenographic changes	Almost complete obliteration of the cartilage space; relative increase in the density of the femoral head; subchondral cyst formation; formation of sequestra; gross deformity of the femoral head; severe spur formation; acetabular sclerosis

**Table 3:** Fluoroscopy and CT scan findings

Case	Fluoroscopy findings (increase in joint space)	CT scan findings with regard to fragments						Additional intraoperative findings
		Size (mm)	Origin	Location	Number	Additional findings	Joint space widening (mm)	
1.	S	2	A	S	1	-	2	-
2.	M	2.5	A	AM	1	-	2	-
3.	S	2	A	SM	1	Undisplaced # P wall of A	2	Cartilage fragments from FH
4.	W	<3	A, FH	AM, PM, SM, IM	>3	-	2	-
5.	M	3-5	FH	PM	3	-	4	Cartilage fragments from FH
6.	S	3	FH	SM	1	-	2	-
7.	M	2	A	PM	2	P labral#	2	-
8.	M	-	-	-	-	Inverted P labrum	3	-
9.	M	<3	FH	M	2	-	3	-
10.	M	2.5	FH	M	1	-	3	-
11.	M	2	A	M	1	-	2	-
12.	W	<3	A	AM, SM, IM	>3	P capsular interposition	2	Cartilage fragments from FH

S: Superior, M: Medial, W: Increase in joint space as a whole, mm: Millimeter, A: Acetabulum, FH: Femoral head, AM: Anteromedial, SM: Superomedial, PM: Posteromedial, IM: Inferomedial, P: Posterior,

# Fracture

**Table 4:** Results

Case	Follow-up (years/months)	Clinical criteria^a^	Roentgenographic criteria^a^
1.	2/5	Excellent	Excellent
2.	2/9	Excellent	Excellent
3.	3	Excellent	Excellent
4.	3/2	Excellent	Excellent
5.	4/2	Excellent	Excellent
6.	4/6	Excellent	Good
7.	5/1	Excellent	Good
8.	5/6	Excellent	Excellent
9.	6/1	Excellent	Excellent
10.	7/8	Excellent	Excellent
11.	7/8	Good	Good
12.	8	Excellent	Good

a Thompson and Epstein clinical and roentgenographic criteria

## RESULTS

**Figure 3:**
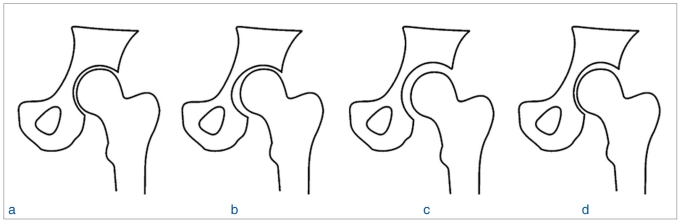
A line diagram showing the radiographic appearance following reduction: (a) Concentric reduction, which was present in 105 patients. (b) Nonconcentric reduction was seen as an increase in medial joint space in seven patients. (c) Nonconcentric reduction was seen as an increase in the joint space as a whole in two patients. (d) Nonconcentric reduction was seen as an increase in the superior joint space in three patients

Twelve of the hundred and seventeen dislocations, which were thought to be simple, resulted in incongruent reduction [Table 1]. The remaining 105 patients had stable concentric reduction, and none of them had radiological evidence of loose bodies; these patients were excluded from the study. Of the 12 patients, 11 were male. The average age was 44.5 years (range: 27–60 years). All patients had posterior dislocation except for one who had an anterior dislocation. Reduction was achieved without difficulty and the joints were stable in all cases. Incongruency was observed at the postreduction fluoroscopic and radiographic analysis. The fluoroscopic assessment of passive motion immediately after reduction showed nonconcentric joint movement in ten patients and increased joint space (when compared to the opposite hip) in two patients. Radiographs showed a break in Shenton’s line with joint space widening on the medial side in seven patients, on the superior side in three patients, and widening of the joint as a whole in two patients and [Figure 3a–d]. CT study of these patients [Table 3] confirmed incongruency and helped in assessing fragment size, location, number, and amount of joint space widening [Figure 1c and 2c]. The size of the fragments was less than 3 mm in nine patients and 3–5 mm in two patients, with the location corresponding to the joint space widening. Six patients had osteocartilaginous fragments originating from the acetabulum, four had fragments from the femoral head, and one patient had fragments from both the femoral head and acetabulum. One patient had an inverted posterior labrum. In 11 patients the loose fragments were extracted within 48 hours [Figure 1d and 2d]; in case 11 [Table 1], extraction was done after 1 month. The average follow-up [Table 4] was 5 years (range: 2 years 5 months to 8 years). All except one patient had excellent result according to Thomson and Epstein clinical criteria. Four patients had early arthritic changes and were graded good and all other had excellent results by Thomson and Epstein roentgenographic criteria. The patient in whom the diagnosis was initially missed had terminal restriction of movements with minimal joint space narrowing and spur formation at the last follow-up. This patient had a good result.

## DISCUSSION

Degeneration of the hip joint following dislocation is one of the most frequently seen long-term complications.^10^,^11^ The factors determining poor outcomes are the energy of the initial violence and the damage to the vascularity of the femoral head at impact, both of which are beyond the control of the surgeon.^2^,^3^,^12^ Poor results can be reduced by reducing the time delay for reduction and by achieving congruent reduction.^7^,^13^,^14^ Congruity is essential for good long-term function of a joint and is particularly important in post-traumatic joints as there is articular damage in addition to incongruity.^7^

A simple dislocation can actually be a fracture-dislocation. The small osteocartilaginous fragments or radiolucent cartilaginous fragments are not visible in roentgenograms and can lead to nonconcentric reduction.^5^,^15^,^16^ In nonconcentric reduction, the fragments inside the joint can abrade the articular surfaces and necessitating surgical removal.^1^,^2^,^7^ Early primary open reduction with removal of all loose fragments that cannot be fixed offers the best prognosis.^1^,^2^ Epstein *et al*.^6^ reviewed 242 dislocations with an average follow-up of about 7 years and concluded that early primary open reduction with removal of all loose fragments of bone and cartilage inside the joint offers the best prognosis. The size of the fragments in the hips in their study ranged from small ones that were not visible on the roentgenogram to some that were 0.25–0.5 cm in diameter. Canale *et al*.^5^ in his review of about 54 dislocations without apparent fractures found that the reduction was not concentric in six patients. They found that what appears to be a non-fracture-dislocation may actually be one, despite the failure to visualize osteochondral fractures on routine roentgenograms. Two patients with nonconcentric reduction in their series were initially missed. They concluded that close scrutiny of postreduction roentgenograms is mandatory. In our review of literature [Table 5] we found that incongruent reductions were often missed after simple dislocations. In all these studies, early identification and open exploration resulted in normal articular function. In our series, except for one patient who was identified late, all patients had excellent clinical outcome.

**Table 5:** Review of literature

Author	Simple dislocations	Nonconcentric reduction	Missed initially	Diagnosed by	Results
Canale *et al*.^5^	54	6	2	Postreduction radiographic analysis	Normal articular function in all
Price *et al*.^21^	3	3	3	Open exploration – 1^a^, CT-2^b^	Early arthritic changes in delayed case
Gennari *et al*.^22^	15	4	-	Open exploration	Normal articular function in all
Our study	117	12	1	Postreduction radiographic and fluoroscopic assessment of passive motion	Clinical outcome E -11; G-1. Radiological outcome E-8, G-4

a Missed in CT and MRI;

b One case retrospectively identified by CT scans; E: Excellent, G: Good

Controversy exists regarding the assessment of adequate reduction. 3D CT in addition to plain films and CT in patients with nonconcentric reduction has emphasized.^2^,^13^ Conversely, a good quality roentgenogram demonstrating concentric reduction rules out loose fragments in the joint.^17^ Our study supports the findings of Frick *et al*.^18^: in all our cases, though the fragment was not visible, the incongruency could be identified by X-rays and confirmed by CT scan. The CT scan was used to identify the location, number and size of the fragment, and the amount of joint space widening. The usefulness of CT scan in assessing the congruity of reduction and any intra-articular fragments has already been demonstrated.^4^ Recent studies have focused on the use of high-resolution techniques, soft tissue windows (400–600 HU), or bone-package images to improve fragment visualization against the soft tissue background.^18^–^20^

MRI scanning is rarely done. It plays a role in the analysis of an incongruent hip in which both plain radiographs and CT images demonstrate unexplained joint widening, in nondisplaced fractures that are not apparent on axial CT scans, and in patients with incarcerated fragments or interposed soft tissues.^1^,^2^,^7^ However, the ‘gold standard’ remains direct visualization of the joint space.^21^,^22^

In our study postreduction fluoroscopic analysis identified all patients with nonconcentric joint movement. In a normal hip, the joint surfaces have a common center of rotation and so the movement of the femoral head over the acetabulum is congruent and the gap between the surfaces is the same on both sides. In incongruent reductions the center of rotation of the femoral head is different when compared to that of acetabulum. Videofluoroscopy of the hip joint while performing passive movements in the flexion-extension axis, abduction-adduction axis, and the rotation axis, identified nonconcentric movements of these hips when compared to the normal hips. This nonconcentric movement was consistent in all our patients and was also confirmed by CT scans and open exploration in our study. The other reliable finding in this group of patients was the increase in joint space in the CT scans.

Our protocol is to assess concentric hip movement immediately after reduction under an image intensifier and to take a good quality postreduction X-ray of both hips and to look for any break in Shenton’s line, widening of joint space, or incongruency in joint space. CT scan is used to find any loose bodies inside the joint and to delineate the anatomy of the fragment if present. In our series, none of the patients had loose bodies either anterior or posterior to the femoral head; this may have been due to the displacement of the fragment deep inside the socket when the joint was tested for stability after reduction. Open exploration and reduction was done in all the patients who had incongruity. Intraoperatively, the hip was dislocated in all cases and the fragments were delivered out, as this has been shown to be the only way to extract all occult fragments.^5^ None of our patients required fixation of the fragments. All the patients had intraoperative roentgenograms, and the movements were checked again under an image intensifier for congruency.

As the degree to which a joint can tolerate incongruity is not clearly defined, it is better to evaluate and treat all reduced hips for incongruity.^7^ Our study emphasizes that even in this era of CT and MRI, postreduction analysis by fluoroscopy and X-rays are a must following reduction of a simple hip dislocation.

## CONCLUSION

The present study shows that nonconcentric reduction can be present in more than 10% of simple dislocations. It is often missed or misdiagnosed as effusion or soft tissue edema or joint laxity. Incongruent reduction is a reliable radiological sign that can be identified by X-rays and fluoroscopy. An immediate joint exploration and removal of the loose bodies is followed by excellent results. Our study supports the view that all patients with dislocation must have a postreduction radiographic study as well as fluoroscopic assessment of passive motion in the operating room immediately after reduction. This will allow for an immediate debridement of the joint and removal of the fragments in these patients without the need of transferring them to the CT suite.
